# Comparing use of BIMA in a Y-graft configuration to BIMA with additional radial artery use during CABG: Two institutional study

**DOI:** 10.1186/1749-8090-10-S1-A105

**Published:** 2015-12-16

**Authors:** D Glineur, RE Shaw, CE Kuschner, F Giovanni, Y Etienne, S Papadatos, M Brizzio, A Zapolanski, JB Grau

**Affiliations:** 1 Department of Thoracic and Cardiovascular Surgery, Cliniques St Luc Bouge, Belgium; 2 Division of Cardiac Surgery, Ottawa Heart Institute, Ottawa, Ontario, Canada; 3 Valley Heart and Vascular Institute, Department of Cardiac Surgery, Ridgewood, NJ, USA; 4 University of Pennsylvania, Department of Surgery, Ridgewood, NJ, USA

## Background/Introduction

Arterial grafting has been demonstrated to confer long-term survival advantages to patients undergoing Coronary Artery Bypass Grafting (CABG). Arterial revascularization may be achieved through the sole utilization of sequential Bilateral Internal Mammary Arteries (BIMA) in a Y-graft construct, or the use of BIMAs with additional radial arteries (RA).

## Aims/Objectives

We assessed the long-term survival of these two approaches.

## Method

Two consecutive series of patients underwent arterial revascularization at two institutions from 2000-2010. In group A, 183 patients underwent CABG with non-sequential BIMA grafting, utilizing the RA for additional targets. In group B, 771 patients underwent solely sequential BIMA grafting in a Y-graft configuration. Patient differences were balanced using a propensity score developed from a logistic regression model with 20 baseline factors. Cox Proportional Hazards Regression was used to adjust for group differences in evaluating survival. In addition, propensity scoring was used to develop two matched cohorts, and survival of these 178 patients was assessed with Kaplan Meier Survival analysis.

## Results

Patients in group B were significantly older (65.7 ± 9 vs. 56.6 ± 10; p < 0.0001) with more diabetes (30.6% vs. 6%; p < 0.0001), CHF (21.0% vs. 2.7%; p < 0.0001), Peripheral vascular disease (21.1% vs. 8.2%; p < 0.0001), renal insufficiency (10.5% vs. 0.0%; p < 0.0001) and dialysis (10.4% vs. 0.0%; p < 0.0001). Group A had a higher proportion of patients with left ventricular ejection fraction less than 50% (26.8% vs. 20.9%; p = 0.046). Both groups had equivalent in-hospital mortality (1.0%), anastomotic sites (mean of 4; p = 0.552) and use of off-pump (both > 90%). In Cox analysis using the propensity score, group B had a trend of improved 14-year survival (92% vs 84%; p = 0.059). Kaplan-Meier analysis of the propensity matched sub-groups showed no statistical significance in 14-year survival (93% vs. 89%; p = 0.101).

## Discussion/Conclusion

Overall there was no statistically significant difference in survival between these two approaches to arterial revascularization. A larger cohort is required to fully compare these techniques.

